# Immune hierarchy among HIV-1 CD8^+^ T cell epitopes delivered by dendritic cells depends on MHC-I binding irrespective of mode of loading and immunization in HLA-A*0201 mice

**DOI:** 10.1111/j.1600-0463.2009.02544.x

**Published:** 2009-11

**Authors:** HENRIK N KLOVERPRIS, INGRID KARLSSON, METTE THORN, SØREN BUUS, ANDERS FOMSGAARD

**Affiliations:** 1 Department of Virology, Statens Serum Institut, Copenhagen SCopenhagen, Denmark; 2 Institute of Medical Microbiology and Immunology, University of CopenhagenCopenhagen, Denmark

**Keywords:** CTL epitopes, HLA-A*0201, immunodominance, dendritic cells

## Abstract

Recent human immunodeficiency virus type 1 (HIV-1) vaccination strategies aim at targeting a broad range of cytotoxic T lymphocyte (CTL) epitopes from different HIV-1 proteins by immunization with multiple CTL epitopes simultaneously. However, this may establish an immune hierarchical response, where the immune system responds to only a small number of the epitopes administered. To evaluate the feasibility of such vaccine strategies, we used the human leukocyte antigen (HLA)-A*0201 transgenic (tg) HHD murine *in vivo* model and immunized with dendritic cells pulsed with seven HIV-1-derived HLA-A*0201 binding CTL epitopes. The seven peptides were simultaneously presented on the same dendritic cell (DC) or on separate DCs before immunization to one or different lymphoid compartments. Data from this study showed that the T-cell response, as measured by cytolytic activity and γ-interferon (IFN-γ)-producing CD8^+^ T cells, mainly focused on two of seven administered epitopes. The magnitude of individual T-cell responses induced by immunization with multiple peptides correlated with their individual immunogenicity that depended on major histocompatibility class I binding and was not influenced by mode of loading or mode of immunization. These findings may have implications for the design of vaccines based on DCs when using multiple epitopes simultaneously.

To meet the high diversity of human immunodeficiency virus type 1 (HIV-1) and to avoid viral cytotoxic T lymphocyte (CTL) escape from the immune system (1), it is desirable to target a broad range of epitopes encoded by distinct proteins in the HIV-1 genome, thereby increasing the coverage of the vaccine. This principle may involve polytope DNA constructs encoding several CTL epitopes (2) or the use of multiple peptides delivered by dendritic cells (DCs) (3–5) and other adjuvants (6). CD8^+^ T cell (CTL) responses generated against antigens that carry several potential CTL epitopes may focus on a minority of epitopes selected by the immune response *in vivo*, a phenomenon known as immunodominance (7). Immunodominance is influenced by antigen processing (7, 8), affinity for the major histocompatibility class I (MHC-I) molecule (9), and direct competition among T cells of the same specificity (10) or different specificities (11).

In this study, we aimed to elucidate the mutual competition among 7 HLA-A*0201 binding HIV-1-derived CTL epitopes (12). These vaccine-relevant CTL epitopes were presented together with a CD4^+^ T-helper peptide either separately or simultaneously on bone marrow-derived DCs and immunized in one or two different anatomic sites of HLA-A*0201 transgenic mice draining to the same or different lymph nodes. We found that the CTL responses focused mainly on the two epitopes with the highest binding affinity to HLA-A*0201.

## Materials and methods

### HLA-A*0201 transgenic mice

The HHD transgenic mice kindly provided by F.A. Lemonnier, Institut Pasteur, Paris, France, express a transgenic monochain histocompatibility class I molecule in which the C-terminus of the human β_2_-microglobulin (β_2_m) is covalently linked to the N-terminus of a chimeric heavy chain (HLA-A2.1 α1-α2, H-2D^b^α3-transmembrane and intracytoplasmic domains). HHD mice are homozygous for the transgene, H-2D^b−/−^ and β_2_m^−/−^ double knockout.

### Peptides and HLA-A*0201-binding

Epitope peptides identified as relevant for HIV-1 vaccination (12, 13) were synthesized by Schafer-N, Copenhagen, Denmark. Peptide-binding affinities to purified HLA-A*0201 molecules were measured using an *in vitro* biochemical binding assay (14). Briefly, pre-oxidized MHC-I molecules when diluted into a reaction buffer containing human β_2_m and the appropriate test peptide, fold rapidly and efficiently only in the presence of the test peptide.

### Generation of mature DCs from bone marrow cells and peptide loading

Bone marrow cells from HHD transgenic mice were kept overnight in culture medium [RPMI 1640 with 10% (v/v) FCS, 1% (v/v) penicillin, streptomycin, 1 ‰ (v/v) 2-mercapta-ethanol] and the non-adhering cells were cultured with GM-CSF, 10 ng/ml, and IL-4, 20 ng/ml (PreproTech Inc., Rocky Hill, NJ, USA). At day 3, fresh culture medium with GM-CSF and IL-4 was supplied. At day 6, the cells were harvested and cultured overnight with GM-CSF, IL-4 and 1 μg/ml of LPS (Sigma, St. Louis, MO, USA). At day 7, the mature DCs were harvested and used for vaccination.

**Fig. 1:**
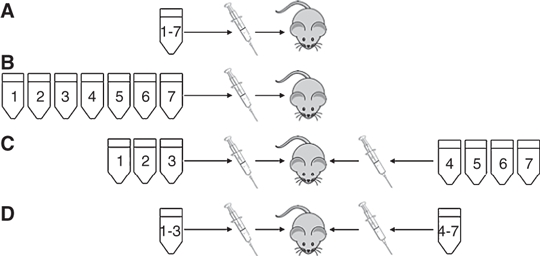
Illustration of four different modes of dendritic cell (DC) loading and immunization. (A) All seven peptides were loaded onto the DCs simultaneously and injected intracutaneously (i.c.) in one flank of the mouse. (B) The same number of DCs was aliquoted in seven vials and pulsed separately with one peptide for each vial and mixed in one syringe followed by i.c. injection in one flank of the mouse. (C) Peptides were loaded separately onto DCs as described in group B, but prior to injection, epitopes ranked 1–3 (Table 1) were pooled and administered in the left flank of the mouse and epitopes ranked 4–7 (Table 1) were pooled and administered in the right flank of the mouse. (D) Epitopes ranked 1–3 and 4–7 (Table 1) were loaded onto DCs in two separate vials followed by i.c. injection in the left and right flanks of the mouse, respectively.

Mature DCs were either loaded with a single peptide for individual peptide immunogenic ranking or loaded with peptides in four different combinations at a concentration of 10 μg/ml/peptide (Fig. 1). Vaccination protocols were performed as follows: (i) all seven peptides were loaded onto the DCs simultaneously, washed thoroughly, resuspended in phosphate-buffered saline (PBS) and injected intracutaneously (i.c.) in one flank of the mouse. (ii) The same number of DCs was aliquoted in seven vials and pulsed separately with one peptide for each vial, then washed thoroughly and mixed in one syringe followed by i.c. injection in one flank of the mouse. (iii) Peptides were loaded separately onto DCs as described in group B, but just prior to injection, epitopes ranked 1–3 according to their individual immunogenicity (Table 1) were pooled and administered in the left flank of the mouse and epitopes ranked 4–7 (Table 1) were pooled and administered in the right flank of the mouse. (iv) Epitopes ranked 1–3 (Table 1) and 4–7 (Table 1) were loaded onto DCs in two separate vials followed by i.c. injection in left and right flank of the mouse, respectively. A CD4^+^ T helper peptide PADRE (15) was included with each CTL epitope peptide to provide necessary CD4 T-cell help. Each mouse received 3 × 10^6^ DCs for individual peptide response or alternatively 1.3 × 10^6^ pulsed DCs for each peptide resulting in 9 × 10^6^ DCs in total for every mouse in the competition experiments.

**Table 1:** Immunogenicity of seven HIV-1-derived HLA-A*0201-binding synthetic peptides

Epitope	Sequence	Rank^1^	IC_50_ (nM)	Specific lysis (%)^2^	SFU/mio splenocytes^3^	IFN-γ^+^TNF-α^+^^4^
Gag_150_^mod^	RLLNAWVKV	1	26	66 ± 1.2	19657 ± 1774	16.6
Gag_433_	FLGKIWPS	2	3	49 ± 2.7	5714 ± 575	12.6
Vif_23_(9V)	SLVKHHMYV	3	168	46 ± 3.6	4217 ± 146	5.0
Pol_606_^mod^	KLGKAGYVV	4	384	36 ± 3.7	4391 ± 370	3.2
Env_67_(2I)	NIWATHACV	5	103	28 ± 0.8	3658 ± 223	3.6
Vif_101_(9L)	GLADQLIHL	6	219	17 ± 0.9	nd	2.5
Vpu_66_^mod^	ALVEMGHHV	7	334	No lysis	nd	2.2

Values represent data from groups of four mice with pooled splenocytes. HIV-1; human immunodeficiency virus type 1; HLA, human leukocyte antigen; SFU, spot-forming unit; IFN-γ, γ-interferon; TNF-α, tumor necrosis factor-α; nd, not determined.

1 Epitopes are ranked according to decreasing immunogenicity.

2 Mean values at effector target ratio 50:1, ± indicate SD of triplicates.

3 Mean values, ± indicate SD of triplicates.

4 Values represent percentage double-positive IFN-γ and TNF-α-producing CD3^+^CD8^+^ T cells from pooled splenocytes (n = 4 mice) in order to obtain sufficient cell counts for optimal analysis.

### HLA-A*0201 transgenic mice immunizations

HHD transgenic mice, 8–12 weeks old, were injected i.c. in the flank with matured DCs pulsed with the relevant HLA-A*0201-binding HIV-1 CTL peptide and a T-helper peptide PADRE as described above (15). DCs were resuspended in 100 μl PBS for each injection. At day 11 after vaccination, mice were sacrificed and the splenocytes were recovered and re-stimulated with the vaccination-peptide as described previously (13). After 5 days, the CTLs were used for flow cytometric analysis or harvested and depleted of NK and B cells using anti-CD49b antibodies and anti-CD19 antibodies (Mylteneyi Biotec, Gladbach, Germany), respectively. Cells were depleted on a column, MACS LS, according to the manufacturer’s protocol. The non-depleted cells were then used directly for cytolytic ^51^Cr-release assays and IFN-γ ELISpot assays.

### Flow cytometry

Splenocytes cultured for 5 days were stimulated for 2 h with 2 μg/ml peptide antigen and subsequently incubated for 5 h with protein transport inhibitor (BD GolgiStop; BD Pharmingen, San Diego, CA, USA) at 37 °C and 5% CO_2_. Thereafter, cells were stored overnight in 4 °C. Due to the low frequency of CD8^+^ T cells in this HHD^+^ transgenic mouse model, CD8^+^ T cells were negatively isolated using a CD8a^+^ T cell isolation kit (Miltenyi Biotec, Teterow, Germany) according to the manufacturer’s protocol, followed by surface staining using anti-CD3-PerCP (BD Pharmingen) and anti-CD8-FITC (BD Pharmingen). Cells were washed and subsequently fixed and permeabilized using the Fix and Perm kit (Caltag Laboratories, Der Grub, Austria) and stained intracellularly with anti-IFN-γ-Allophycocyanin (APC) (BD Pharmingen) and anti-tumor necrosis factor-α (TNF-α)-Phycoerythrin (PE) (BD Pharmingen). Cells were washed and resuspended in staining buffer prior to immediate flow analysis. Data were acquired on a BD LSRII instrument using FACSdiva software (BD Biosciences) and analyzed with FACSdiva or FlowJo software (TreeStar, Ashland, OR, USA).

### IFN-γ enzyme-linked immunospot (ELIspot) assay

A standard IFN-γ ELISpot assay was used as described previously (13). Cells, with titration of cells ranging from 300 000 to 18 750 each performed in triplicate, were stimulated with 10 μg of antigen/ml for 18 h of incubation at 37 °C, 5% CO_2_. Spot-forming units were quantified using an automatic system ELR02 ELISPOT reader (Autoimmun Diagnostika GmbH, Strassberg, Germany) and analyzed with AiD3.1 S.R software.

### Cytotoxic T lymphocyte chromium release assay

A standard cytotoxic ^51^Cr-release assay was used as described previously (13). Briefly, HHD-EL4S3^−^Rob target cells (16) were mixed with peptide loaded splenocyte effector cells at effector:target cell ratios (E:T) of 100:1, 50:1, 25:1 and 12.5:1 in either triplicates or six replicates. ^51^Cr-release was measured using a microplate scintillation counter (Topcount, NXT; Packard, Boston, MA, USA). Spontaneous and total ^51^Cr-release was measured by adding culture medium or a detergent (Triton X-100) 2% v/v, respectively. The percentage of specific lysis was calculated as 100 × (experimental release − spontaneous release)/(total release − spontaneous release). Spontaneous and total release was in the range of 1200 and 9000 counts per minute, respectively.

## Results

### Immunogenicity of individual peptides

To evaluate the individual immunogenicity of the minimal HIV-1-derived HLA-A*0201-binding CTL epitopes we immunized HLA-A*0201 tg mice with DCs pulsed with the individual CTL epitope. We used a standard specific cell killing assay (chromium release), confirmed by intracellular cytokine staining (ICS) and ELISpot assays (Table 1). We found that each of the seven CTL epitopes tested exhibited differential immunogenicity *in vivo*, with the two Gag epitopes Gag_433_ and Gag_150_^mod^ being the most immunogenic CTL epitopes. The immunogenicity was comparable with the HLA-A*0201 binding affinity where the two Gag peptides bind better than 100 nM and the remaining peptides bind in the intermediate 100 nM < IC_50_ < 500 nM range (Table 1). For further *in vivo* competition experiments when immunizing multiple CTL epitopes simultaneously we ranked the seven CTL epitopes with respect to immunogenicity measured by functional lytic activity (Table 1).

### *In vivo* competition is not reduced by pulsing epitopes separately on DCs

**Fig. 2:**
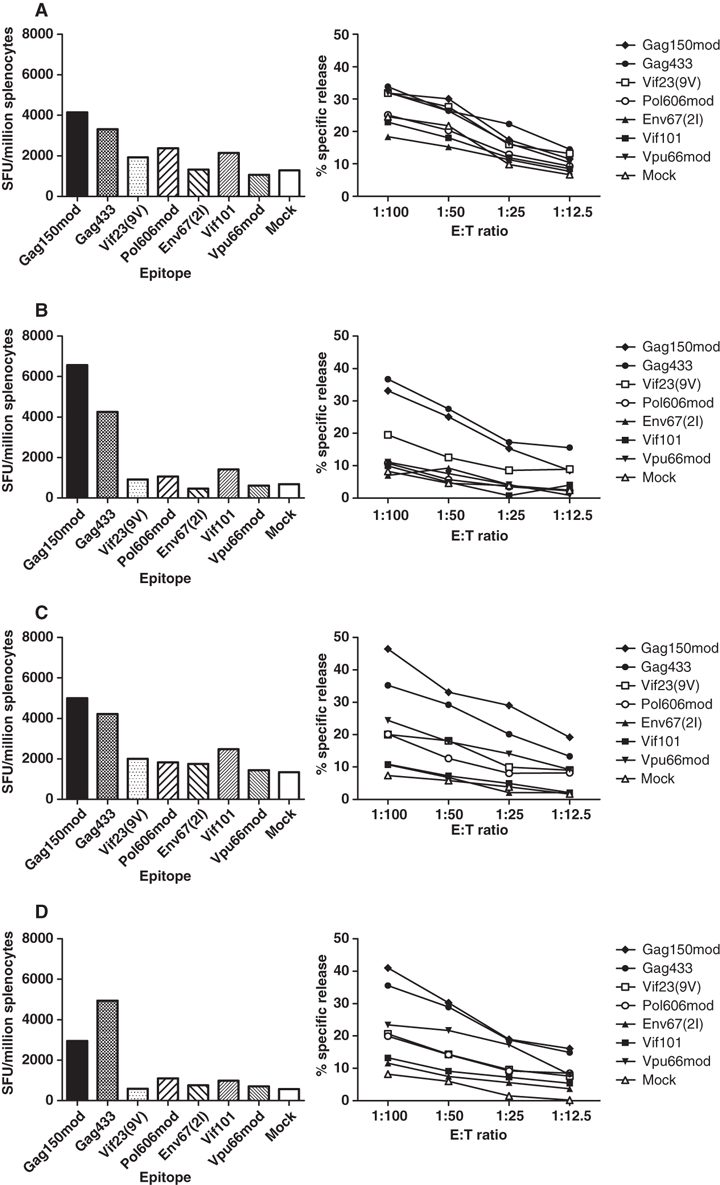
Two of seven epitopes dominate the T-cell responses. Chromium release cytotoxic T lymphocyte (CTL) assay and IFN-γ ELISpot assay were used to measure the breadth and magnitude of T-cell responses. (A) (B) (C) and (D) are illustrated in Fig. 1A–D, respectively, and explained in the legend to Fig. 1. Groups of six mice were used for immunization and pooled splenocytes were tested against all seven CTL epitopes and one irrelevant negative control peptide (mock). SFU, spot-forming unit. One representative experiment of three is shown.

We examined whether immune competition would occur and could be changed by presenting epitopes either separately or simultaneously on bone marrow-derived DCs prior to immunization in HLA-A*0201 transgenic mice. This was done by pulsing all seven peptides in one vial vs pulsing all seven peptides in seven individual vials of DCs as illustrated in Fig. 1A,B, respectively. These experiments were repeated three times. By simultaneous immunization, a hierarchy was established where Gag_150_^mod^ and Gag_433_ responses dominated (Fig. 2). Irrespective of whether the epitopes were pulsed separately before pooling, or pulsed together onto DCs, the resulting *in vivo* hierarchy was virtually unchanged as measured by cytolytic and ELISPOT assays. One representative experiment is shown in Fig. 2. These data were confirmed in another experiment using IFN-γ, TNF-α and IL-2 ICS (data not shown). In some experiments with individual pulsing or grouping according to the ranking shown in Table 1, up to three additional epitopes showed a weak response in some assays, as Vif_23_ in Fig. 2B. However, these weaker responses were not consistent.

### *In vivo* competition is not reduced by separating epitopes and immunizing different lymphoid compartments

We then hypothesized whether we could overcome the immunodominance of Gag_433_, Gag_150_^mod^ and Vif_101_, ranked 1–3 in Table 1, by homing DCs loaded with more dominant CTL epitopes and DCs loaded with less dominant CTL epitopes, to the same or different lymphoid compartments, respectively. We immunized dominant and less dominant CTL epitopes separately in the right and left flank of the mouse, respectively, as indicated in Fig. 1C,D. Administration of dominant and less dominant peptides in different lymphoid compartments did not increase the breadth or the magnitude of CTL responses *in vivo* (Fig. 2C,D). Again, the epitopes Gag_150_^mod^ and Gag_433_ dominated the responses in three individual experiments (Fig. 2).

## Discussion

Immunodominance in the context of CTL epitopes is influenced by a number of factors including the T-cell precursor repertoire (10), T-cell access to the antigen-bearing APC (10), TCR affinity for the antigen (17), antigen affinity for MHC-I and the processing of antigen (7).

In this study we evaluated whether multi-epitope immunization would change the immunogenicity of individual epitopes and whether different modes of DC immunization would change or reduce any immunodominance and thereby broaden the T-cell responses. We used seven HIV-1-derived CTL epitopes each found to be immunogenic by peptide immunization in Freund’s incomplete adjuvant (12) and by individual peptide DC immunization (Table 1). When peptides were delivered simultaneously on the same DC, separate DCs and/or distributed to different lymphoid compartments the CTL response focused mainly on two epitopes, Gag_150_^mod^ and Gag_433_. Interestingly the remaining epitopes Vif_23_, Pol_606_^mod^, Vpu_66_^mod^, Vif_101_ and Env_67_ showed weaker and inconsistent responses compared to their immunogenicity when delivered with DC alone. This phenomenon was virtually unchanged using the different modes of immunization with seven epitopes.

The observed immunodominance for Gag_150_^mod^ and Gag_433_ cannot be explained by intracellular preference for antigen processing, because all peptides were loaded onto mature DCs in a pre-processed form. Instead, the different epitope HLA-A*0201-binding affinities (Table 1) may explain the immunodominance pattern, where Gag_150_^mod^ and Gag_433_ had the highest binding affinities to HLA-A*0201 (IC_50_ < 100 nM). This is in line with previous findings that also relate high MHC-I affinity and immunodominance (7, 18) where good immunity is induced by peptide epitopes binding better than 100 nM, and no immunogenicity by MHC-I processing either in the intermediate range 100 < IC_50_ < 500 nM or in the no MHC-I binding range IC_50_ > 500 nM (9, 12).

Peptide competition for the MHC-I molecule may impact on the output response *in vivo* (18, 19). However, peptide competition for the APC does not seem to obscure the dominance pattern in our system, where approximately the same CTL epitopes stimulate responses when antigens are delivered on the same DC or on separate DCs. These findings are in contrast to previous reports, which suggest that T-cell competition is decreased when antigen is presented on different APCs (11, 20). This may be due to the relatively high numbers of DCs used for immunization, where 1.3 × 10^6^ pulsed DCs for each peptide resulting in a total of ∼9 × 10^6^ DCs are injected into each mouse. Instead, it appears in our model that TCR affinity for the antigen largely impacts the immune hierarchy. The very low number of naïve CD8^+^ T cells specific for a given antigen in the very early phase of a primary response makes competition at this stage unlikely (21). However, as the proliferation of these specific cells increases, competition for persistent TCR stimuli will be established, especially among cells of the same clone and avidity occupying the same niche in the lymphoid compartment (22), possibly explaining the cross-competition observed in this study.

In a recent phase I trial of therapeutic immunization using the same minimal CTL epitope peptides and DC in 12 HLA-A0201^+^ HIV-1^+^ individuals, we used an immunization strategy similar to D in Fig. 1. Although different immune reaction patterns were observed in individual patients, all epitopes were immunogenic, inducing new immunity in at least one patient as measured by ICS and/or pentamer staining (5). The most frequent targeted epitope was Pol_606_^mod^ followed by Vif_23_, Gag_433_, Env_67_, Gag_150_^mod^, Vif_101_ and Vpu_66_^mod^. The fact that the immune hierarchy seen in HLA-A2tg mice did not translate into humans may be due to several reasons: the assays used for evaluation were different, in humans we used ICS and pentamer staining and in mice we used cytotoxic ^51^Cr-release and IFN-γ ELISPOT. The HLA-A2tg mice are homozygous for the HLA-A*0201 transgene and H-2D^b−/−^ knockout, whereas although all individuals in the human study were HLA-A0201^+^, epitope promiscuity and affinity to other HLA alleles likely influence the immune hierarchy (23). Moreover, HIV-1^+^ individuals have, before this therapeutic vaccination, been exposed to these HIV-1-derived epitopes.

Our results show that the two epitopes Gag_150_^mod^ and Gag_433_ established themselves as dominant in the context of the seven epitopes tested, whereas the remaining five epitopes established themselves as subdominant in the multi-epitope context chosen. It appears that binding affinity to MHC-I is the determining factor in the established hierarchy that could not be avoided or changed by the different modes of immunizations used here. Although, HLA-A0201^+^ HIV-1-infected humans and HLA-A*0201tg mice may react differently. These findings may have implications for DC-based vaccine strategies that intend to optimize administration of multiple antigens concurrently.
